# Patient First Strategies for Reducing Inequities in HAI Prevention

**DOI:** 10.1017/ash.2024.207

**Published:** 2024-09-16

**Authors:** Lisa Stancill, Emily Sickbert-Bennett Vavalle, Lauren DiBiase

**Affiliations:** UNC Health

## Abstract

**Background:** Inequities in healthcare-associated infections (HAI) incidence and prevention measures are critically important to understand (Chen,2021). While evaluations are beginning to characterize these disparities by infection type (Gettler, 2023), our work expands this by characterizing disparities by prevention strategies. By better understanding how evidence-based prevention strategies are implemented at the patient level, infection preventionists and hospital epidemiologists can better design strategies that provide equitable care to all patients. **Methods:** Beginning January 2023, gender, race, ethnicity, spoken language, and age group fields were added to daily chlorhexidine gluconate (CHG) treatment and C. difficile test order compliance data captured via electronic medical record. In July 2023, fields on recorded race, ethnicity, and gender were added to well-established foley and vascular access real-time peer audit tools that are used by infection preventionists (IPs). Each prevention strategy variable was summarized by demographic variables and differences in compliance were measured using chi-square tests. **Results:** 899 vascular audits and 420 foley audits were completed by IPs between July – December 2023. In 2023, there were 114,066 opportunities for CHG Treatment and 1,991 C. difficile test orders. Missing data varied by metric but ranged from 0-60%. Statistically significant differences by race were found in 3 of 8 components (i.e., intact seal, secured catheter and absence of dependent loop) in the foley audit (p < 0 .01) and compliance with C. difficile test ordering (p < 0 .01). No differences in race were found in vascular access audits or CHG treatment. No differences in gender or ethnicity were noted in foley, vascular access audits, CHG treatment compliance, or C. difficile testing. Differences in gender and age were found in CHG treatment compliance (p < 0 .001). **Conclusions:** By focusing more on patient level process measures rather than only presenting stratified outcomes data, we can identify targeted opportunities for improvement in health equity before our patients develop an HAI. Further evaluations should also focus on assessing the clinical relevance of statistical findings to better inform intervention strategies. Separately, efforts are needed to improve completeness and integrity of demographic data in the electronic medical record.

